# Emergence of Unconventional Magnetic Order in Strain‐Engineered RuO_2_/TiO_2_ Superlattices

**DOI:** 10.1002/advs.77914

**Published:** 2026-09-27

**Authors:** Seung Gyo Jeong, Seungjun Lee, Jin Young Oh, Bonnie Y. X. Lin, Anand Santhosh, Sehwan Song, Sungkyun Park, James M. LeBeau, Alexander J. Grutter, Woo Seok Choi, Tony Low, Valeria Lauter, Bharat Jalan

**Affiliations:** ^1^ Department of Chemical Engineering and Materials Science University of Minnesota−Twin Cities Minneapolis Minnesota USA; ^2^ Now at Department of Physics Hankuk University of Foreign Studies Yongin Republic of Korea; ^3^ Department of Electrical and Computer Engineering University of Minnesota−Twin Cities Minneapolis Minnesota USA; ^4^ Now at Department of Applied Physics Kyung Hee University Yongin Republic of Korea; ^5^ Department of Physics Sungkyunkwan University Suwon Republic of Korea; ^6^ Department of Materials Science and Engineering Massachusetts Institute of Technology Cambridge Massachusetts USA; ^7^ Department of Physics Pusan National University Busan Republic of Korea; ^8^ NIST Center for Neutron Research National Institute of Standards and Technology Gaithersburg Maryland USA; ^9^ Neutron Scattering Division Oak Ridge National Laboratory Oak Ridge Tennessee USA

**Keywords:** magnetism, molecular beam epitaxy, polarized neutron reflectometry, RuO_2_, strain engineering, symmetry modulation

## Abstract

The spin ordering in RuO_2_ remains a highly debated topic, owing to its elusive nature, with reports ranging from a nonmagnetic ground state to signatures of unconventional magnetic order. Here we provide the first unambiguous and direct evidence of unconventional magnetism in epitaxial, fully strained RuO_2_/TiO_2_ superlattices on a TiO_2_ (110) substrate by using hybrid molecular beam epitaxy. Polarized neutron reflectometry reveals a finite magnetic moment localized within the compressively strained RuO_2_ layers, consistent with predictions obtained from first‐principles calculations. Complementary density functional theory calculations and x‐ray photoemission spectroscopy measurements show that epitaxial strain drives the Ru 4*d* states toward the Fermi level, triggering a Stoner‐type instability that stabilizes uncompensated magnetic order. These unique results reveal that RuO_2_ exhibits unconventional magnetic states under epitaxial strain, which are not accessible in bulk RuO_2_, and establish strain engineering as a powerful route to uncover and control magnetic phases in RuO_2_ and related oxides.

## Graphical abstract

Epitaxial strain activates unconventional magnetism in rutile RuO_2_, absent in bulk or relaxed RuO_2_. Polarized neutron reflectometry with atomic‐layer resolution directly reveals finite magnetization confined to RuO_2_ layers in fully strained RuO_2_/TiO_2_ superlattices on TiO_2_ (110). Theory and spectroscopy show that strain shifts Ru 4*d* states toward the Fermi level, triggering a Stoner instability.

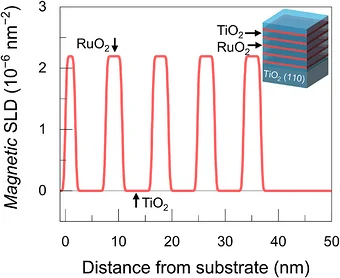

## 1 Introduction

The magnetic state of RuO_2_ has remained a subject of vigorous debate. Rutile RuO_2_ has been proposed as a *d*‐wave altermagnet [1, 2], characterized by rotated spin densities, staggered spin polarizations, and a Néel vector aligned along the [001] crystallographic direction. Early neutron and resonant x‐ray diffractions reported a magnetic Bragg peak at the (100) reflection in bulk crystals and epitaxial films [3, 4], suggesting a small, antiferromagnetically (AFM) ordered moment persisting above room temperature. Yet, later studies demonstrated that this reflection could arise from the intrinsic charge anisotropy of the rutile lattice [5, 6], casting doubt on these earlier interpretations. Complementary muon spin rotation/relaxation (µSR) measurements have also produced conflicting results—initial experiments detected no magnetic moments in bulk crystals and sputtered epitaxial films [7, 8], whereas more recent low‐energy µSR with approximately 10 nm depth resolution revealed enhanced muon asymmetry near the surface in epitaxial films [9]. Angle‐resolved photoemission spectroscopy (ARPES) has likewise yielded inconsistent observations across sample geometries and different synthesis techniques [10, 11, 12, 13, 14, 15].

**FIGURE 1:**
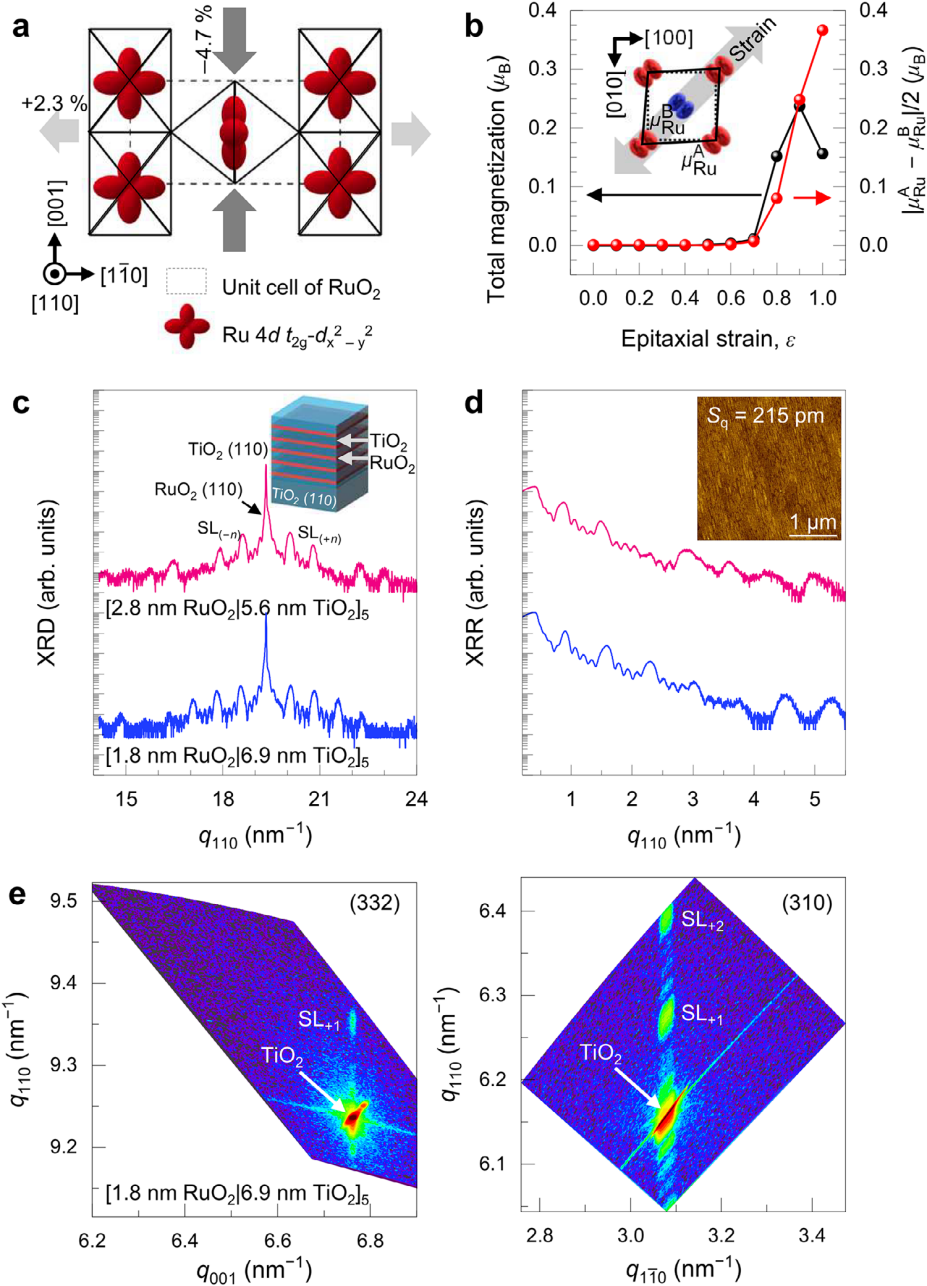
Atomically well‐defined and fully strained RuO_2_/TiO_2_ (110) superlattices grown by hybrid molecular beam epitaxy. (a) Schematic illustration of strain‐induced modifications of the Ru 4*d* orbital states in strained RuO_2_. In rutile RuO_2_, the edge bonding states of *t*_2g_ orbitals could be modulated significantly by [001]‐direction strain. (b) Evolution of total magnetization (left, black symbols) and Néel order (right, red symbols) as a function of epitaxial strain (*ε*), demonstrating the emergence of magnetism and the presence of an uncompensated magnetic moment under strained conditions. Especially for *ε* = 1, the larger Néel order compared to total magnetization indicates the existence of uncompensated magnetization. (c) High‐resolution XRD *θ–*2*θ* and (d) XRR scans of the RuO_2_/TiO_2_ (110) superlattice thin films. Multiple superlattice satellite peaks (SL _±_ *_n_*) with Kiessig fringes in *θ–*2*θ* scans and Laue oscillations in XRR scans indicate the intended periodicity and atomically sharp interfaces. The XRD *θ–*2*θ* curves are vertically offset by 10^7^ and the XRR curves by 10^6^ for clarity. The inset of (d) shows an atomic force microscopy image revealing an atomically smooth surface with a root‐mean‐square roughness of *S*_q_ = 215 pm. (e) XRD RSM scans around the (332) and (310) TiO_2_ reflections show identical in‐plane reciprocal space wave vectors between the superlattice and substrate, indicating a fully strained state.

The diverging experimental reports highlight the importance of synthesis parameters such as sample geometry, dimensionality, defect chemistry, and epitaxial strain. While RuO_2_ bulk is predominantly reported to be nonmagnetic [16, 17, 18, 19, 20], RuO_2_ thin films show a significantly broader spectrum of magnetic responses [12, 21, 22, 23, 24, 25, 26, 27, 28, 29, 30, 31, 32, 33, 34, 35, 36, 37, 38, 39, 40, 41, 42]. These findings imply that the reduced dimensionality and/or interfacial constraints may substantially influence the structural, electronic, and magnetic ground states. Among these factors, epitaxial strain—owing to the lattice mismatch between RuO_2_ films and TiO_2_ (110) substrates—plays a particularly crucial role (Figure 1a,b and Figure S1). Especially, compressive strain along [001] can shift the Ru 4*d* states toward the Fermi level (Figure S1), enhancing the Stoner instability [22, 23], analogous to strain‐driven magnetism in monolayer SrRuO_3_ [43]. Fully strained (110)‐oriented films on TiO_2_ (110) further break the two‐fold spin‐rotation symmetry generated by four‐fold rotation combined with a half translation, giving rise to an uncompensated magnetic order, where the magnetic moments do not fully cancel and produce a small net magnetic moment (weak ferromagnetism (FM); Figure 1b) [21, 22, 23].

Even small variations in strain state across differently grown samples can therefore yield qualitatively distinct magnetic responses, underscoring the need for a robust observation of magnetism in *fully strained* RuO_2_ films on TiO_2_ (110) substrates. Yet, a direct magnetization measurement of such films has remained elusive. Although heterostructures incorporating a ferromagnetic layer have been explored to probe the magnetism of RuO_2_ via spin torque or exchange bias measurements [25, 26, 27, 28, 29, 30, 31, 32, 33, 34, 35, 36, 37, 38, 39, 41, 44], such approaches are inherently affected by proximity effects between the ferromagnetic layer and RuO_2_, making it difficult to isolate the magnetization response of RuO_2_ itself. Moreover, experimental investigations on ultrathin, fully strained films are highly limited, likely because disorder and defects suppress the intrinsic metallicity and any emergent magnetic behavior. Hybrid molecular beam epitaxy (MBE) grown, fully strained RuO_2_/TiO_2_ (110) films have shown a robust metallicity and crystalline quality of the RuO_2_ layer with several promising signatures of magnetism, including time‐reversal symmetry breaking in optical second‐harmonic generation [21], magneto‐optical responses [21], anomalous Hall effects [23], and spin‐split bands in spin‐ARPES [12]. Notably, the observation of an anomalous Hall effect under magnetic fields applied along both out‐of‐plane [110] and in‐plane [001] directions suggests the possible spin canting in this system [23]. However, these experiments do not directly quantify net magnetization; therefore, a direct measurement of the magnetic order is still highly needed.

Here, we directly examine the uncompensated magnetic order in fully strained RuO_2_ superlattices using polarized neutron reflectometry (PNR). PNR offers sub‐nm depth resolution and isolates the layer‐selective magnetic asymmetry characteristic of uncompensated order [45, 46, 47, 48, 49, 50, 51, 52], separating magnetic scattering from structural contributions. To maximize magnetic contrast, we synthesized atomically well‐defined RuO_2_/TiO_2_ superlattices on TiO_2_ (110) substrates using hybrid MBE [12, 21, 22, 23, 49], achieving high signal‐to‐noise ratios in PNR experiment at the superlattice peaks while maintaining a fully strained state. By combining density functional theory (DFT) with X‐ray photoelectron spectroscopy (XPS), we reveal that epitaxial strain pushes the Ru 4*d* states toward the Fermi level, inducing Stoner instability. We observe clear signatures of uncompensated magnetic order in the strained RuO_2_ layers—reproduced across multiple measurements and different samples—in agreement with DFT calculations. These results demonstrate that epitaxial strain stabilizes the uncompensated magnetic order in RuO_2_ and that the magnetism is not confined only to the surface. Our unique results unambiguously demonstrate how atomic‐scale synthesis and strain engineering lead to unconventional magnetic states otherwise inaccessible in bulk form, providing a robust pathway for designing strain‐engineered RuO_2_ for next‐generation spintronic and quantum technologies.

## 2 Results and Discussion

To obtain high signal‐to‐noise ratios at the superlattice peaks while maintaining epitaxial strain, we synthesized epitaxial RuO_2_/TiO_2_ (110) superlattice structures using hybrid MBE. We designed two superlattice variants with RuO_2_ layer thicknesses of 2 and 3 nm—both below the ∼4 nm critical thickness for strain relaxation [21], corresponding to the thickness regime where strain‐induced structural, electronic, and magnetic phenomena have previously been observed [12, 21, 22, 23, 24]—while keeping the overall supercell thickness near 8.5 nm with five repetitions. Such a design optimally places the primary superlattice reflection peak at a low out‐of‐plane wave vector (*q*_110_), ensuring that multiple superlattice peaks fall within the accessible *q*‐range for PNR. Streaky RHEED patterns after growth with pronounced Kikuchi lines along both the ⟨1$\bar{1}$0⟩ and ⟨001⟩ azimuths confirm excellent crystalline order and smooth surfaces (Figure S2).

High‐resolution x‐ray diffraction (XRD) *θ–*2*θ* scans and x‐ray reflectivity (XRR) measurements verify the (110)‐oriented superlattice structure (Figure 1c,d). The TiO_2_ (110) Bragg peak appears at *q*_110_ = 19.3 nm^−1^ with a distinct shoulder originating from the RuO_2_ layers in both samples. Multiple superlattice satellites (SL _±_ *_n_*) confirm the well‐defined RuO_2_/TiO_2_ periodicity, and the periodicities match the designed supercell dimensions. Pronounced and multiple Laue oscillations between Bragg reflections further indicate atomically sharp interfaces. Supercell thicknesses for two different samples extracted from XRD (8.6 and 8.5 nm, respectively) agree with values obtained from PNR fitting (discussed below). Based on PNR and XRR, we assign the layer sequences as [1.8 nm RuO_2_|6.9 nm TiO_2_]_5_ and [2.8 nm RuO_2_|5.6 nm TiO_2_]_5_ for the two samples, respectively. In the XRR scans, the multiple reflectivity oscillations of the superlattice persist up to *q*_110_ = 5.5 nm^−1^, demonstrating the atomically smooth surface and low interface roughness of both superlattices. A representative atomic force microscopy image for [1.8 nm RuO_2_|6.9 nm TiO_2_]_5_ sample further confirms atomically smooth surfaces, with a root mean square (RMS) roughness (*S*_q_) of 0.2 nm (Inset of Figure 1d).

XRD reciprocal space maps (RSMs) around the TiO_2_ (332) and (310) reflections confirm that both in‐plane crystal orientations ([001] and [1$\bar{1}$0]) remain fully strained (Figure 1e), revealing emerging a nontrivial magnetic state in RuO_2_ layers (Figure 1b). For RuO_2_/TiO_2_ (110), the lattice mismatches between RuO_2_ and TiO_2_ are −4.7% along [001] and +2.3% along [1$\bar{1}$0] (Figure 1a). In single‐layer RuO_2_ films, the RuO_2_ Bragg reflection peak largely overlaps with that of the TiO_2_ substrate, making it difficult to determine the strain state using laboratory‐based XRD. However, in the superlattice geometry, the appearance of well‐defined superlattice satellite peaks provides direct evidence of the periodic RuO_2_/TiO_2_ stacking and contains structural information from both constituent layers, confirming that the RuO_2_ and TiO_2_ layers remain coherently strained to the TiO_2_ (110) substrate. Furthermore, Ru *L*_3_‐edge XAS measurements reveal that Ru remains predominantly in the +4 oxidation state in both the superlattice and single‐layer RuO_2_ films [23], indicating the absence of substantial Ru reduction or large oxygen deficiency [53] and supporting strain as the primary parameter associated with the emergent magnetic behavior.

Our DFT calculations indicate that the fully strained state shifts the Ru 4*d* valence states closer to the Fermi level, thereby promoting the stabilization of a magnetic ground state following the Stoner model (Figure S1a). This strain‐induced band shift is consistent with our XPS results on RuO_2_/TiO_2_ (110) films revealing that the Ru 4*d* valence‐band peak systematically moves toward the Fermi level with decreasing thickness (increasing strain) (Figure S1b). The DFT calculations further reveal an uncompensated magnetic order in a fully strained epitaxial RuO_2_/TiO_2_ (110) system (Figure 1b).

**FIGURE 2:**
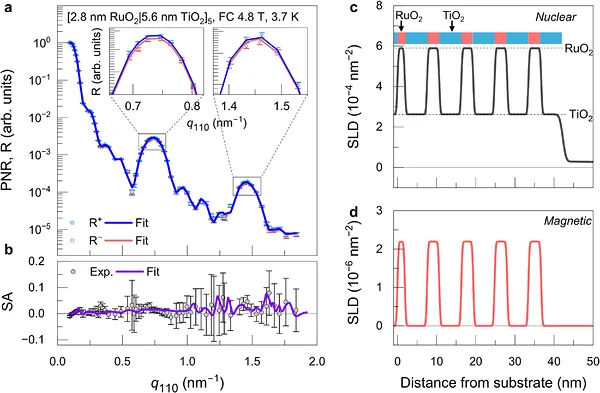
Uncompensated magnetic order in fully strained RuO_2_/TiO_2_ superlattices revealed by polarized neutron reflectometry. (a) Spin‐resolved PNR spectra (R^+^ and R^−^) of the RuO_2_/TiO_2_ superlattice, together with the corresponding fits. Measurements were performed at 3.7 K under a 4.8 T magnetic field applied along the [001] direction. Insets show magnified views of the PNR spectra near the superlattice Bragg peaks, where the splitting between R^+^ and R^−^ becomes evident. (b) Spin asymmetry, SA, along with the model fits. (c) Depth profiles of the nuclear and (d) magnetic scattering length densities (SLDs) used for reflectivity modeling. Horizontal dashed lines indicate the maximum SLD values and serve as guides to the eye. The inset in (c) provides a schematic illustration of the superlattice stack.

The PNR measurements directly confirm the presence of net magnetization in the fully strained RuO_2_ layers of the superlattices (Figure 2). Figure 2a shows the PNR spectra of the [2.8 nm RuO_2_|5.6 nm TiO_2_]_5_ superlattice measured at 3.7 K under a 4.8 T magnetic field applied along the [001] direction. R^+^ and R^−^ denote the PNR spectra spectrum measured with the neutron spin parallel and antiparallel to the applied magnetic field, respectively. To suppress potential domain formation and enhance magnetic contrast, the sample was field‐cooled (FC) from room temperature in a 4.8 T field along [001], and measurements were performed at a temperature (*T*) < 4 K, which is lower than the theoretically predicted magnetic anisotropy energy scale (∼13 K) [23]. This FC protocol minimizes domain‐related averaging effects and provides a clearer measurement of the net magnetic response. The insulating TiO_2_ layers of the superlattices are thick enough that any direct exchange coupling between RuO_2_ layers should be strongly suppressed [54], so the observed signal is interpreted as a layer‐confined RuO_2_ magnetization aligned by the applied field. Similar to XRR, PNR exhibits well‐defined Kiessig fringes and superlattice peaks at *q*_110_ = 0.75 and 1.48 nm^−1^, reflecting excellent crystallinity and supercell thickness. Magnified views near the Bragg reflections (inset of Figure 2a) reveal a clear separation between R^+^ and R^−^ beyond the experimental uncertainties, demonstrating the existence of uncompensated magnetic order in the fully strained RuO_2_/TiO_2_ superlattices. To further confirm reproducibility, we measured two different superlattice samples—[1.8 nm RuO_2_|6.9 nm TiO_2_]_5_ and [2.8 nm RuO_2_|5.6 nm TiO_2_]_5_—within a single beamtime session (Figure S3), and performed additional measurements on the [1.8 nm RuO_2_|6.9 nm TiO_2_]_5_ sample during a separate beamtime (Figure S4). All three independent datasets consistently show robust splitting between R^+^ and R^−^ at the superlattice peaks, firmly establishing the presence of uncompensated magnetic order. In contrast, no magnetic splitting is observed near the superlattice peaks when the measurement is performed along the [1$\bar{1}$0] direction (Figure S5), suggesting that the uncompensated magnetic component lies along the [001] direction. We also note that the PNR method is three‐dimensional depth‐resolved vector magnetometry sensitive to the in‐plane magnetization vector [55, 56]. To separate the nuclear from the magnetic scattering, the data are presented as the spinasymmetry (SA) ratio SA = (R^+^(*q*_110_) − R^−^(*q*_110_))/(R^+^(*q*_110_) + R^−^(*q*_110_)), as depicted in Figure 2b, highlighting the splitting near the superlattice reflections, where the reflectivity signal is maximal, and the statistical uncertainties are minimal. The SA near the critical edge at *q*_110_ = 0.16 nm^−1^ also exhibits a small but robust spin splitting exceeding statistical uncertainties, further supporting the presence of a net magnetization in the superlattices. In contrast, the small SA signal away from the superlattice peaks is below the measurement uncertainty, precluding a detailed spin‐dependent analysis in these regions.

To quantify the magnetic signal, we fitted the PNR spectra using a minimal superlattice model (see Figure S6 and Table S1, for a more detailed model analysis), intentionally avoiding overly complex parameterizations. The resulting fits (solid lines in Figure 2a,b) show good agreement with the experimental data, particularly in the vicinity of the superlattice reflections where the magnetic contrast is most pronounced. The 𝜒 [3, 4] value of 2.50 further indicates that the deviations between the experimental data and the simulations remain within statistical expectations. Figure 2c,d presents the nuclear and magnetic scattering length densities (SLDs) extracted using Ref1D, revealing an atomically well‐defined periodic structure and a finite uncompensated magnetic moment confined to each RuO_2_ layer. The nuclear SLD of the RuO_2_ layers is (5.90 ± 0.01) × 10^−4^ nm^−2^, in excellent agreement with the theoretical bulk RuO_2_ density of 5.88 × 10^−4^ nm^−2^. The magnetic SLD of the RuO_2_ layers is 2.19 × 10^−6^ nm^−2^, corresponding to the averaged magnetization of (0.0219 ± 0.0039) *µ*_B_ f.u.^−1^. To test alternative scenarios, we simulated several possible magnetic configurations, including conventional FM in RuO_2_ (Figure S7a) and magnetism residing in the TiO_2_ layers (Figure S7b). These models produce signatures that clearly differ from the experimental data; in particular, a magnetic TiO_2_ layer would yield an opposite sign of spin splitting at the superlattice peaks (Figure S7b), which is incompatible with our observations. PNR fits obtained for another sample [1.8 nm RuO_2_|6.9 nm TiO_2_]_5_ superlattice also confirm the presence of uncompensated magnetic order in each RuO_2_ layer (Figure S6b). Notably, the difference in spin splitting between the first and second superlattice peaks suggests a possible interfacial magnetism in RuO_2_ (Figure S8), which we attribute to the reduced RuO_2_ thickness. Independent magnetization measurements on a [2 nm RuO_2_|1 nm TiO_2_]_5_ superlattice, which has a different total thickness but a comparable strain state [21] to the superlattice used for the PNR measurements, further support the presence of weak ferromagnetism by revealing a small but reproducible magnetic hysteresis in the RuO_2_/TiO_2_ superlattice, whereas no comparable signal is observed in a TiO_2_ reference film (Figure S9).

**FIGURE 3:**
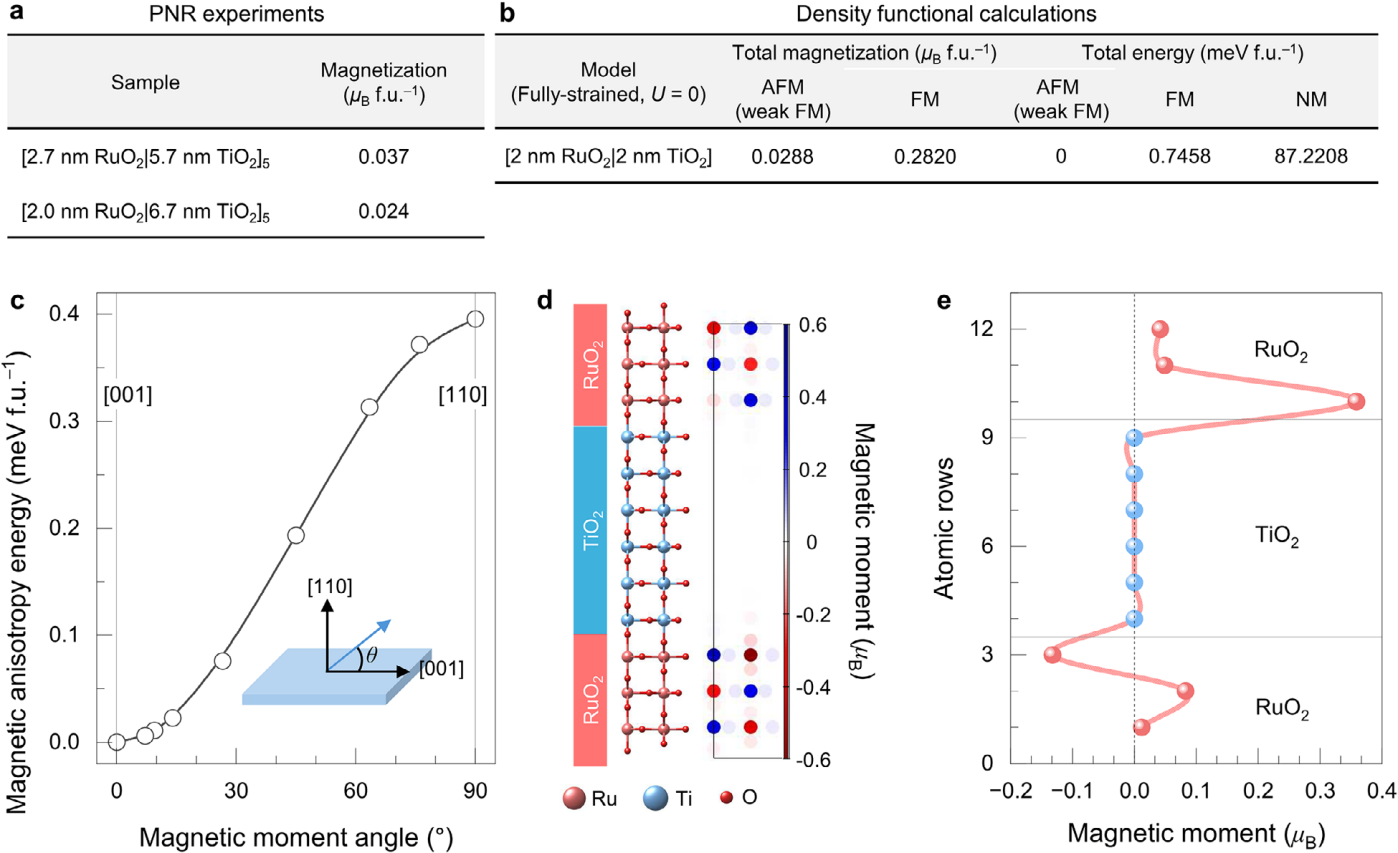
Experimental magnetization and theoretical description of uncompensated magnetic order in strained RuO_2_/TiO_2_ superlattices. (a) The magnetization values extracted from PNR measurements for two RuO_2_/TiO_2_ superlattice samples, demonstrating the presence of weak yet finite magnetic moments in the fully strained RuO_2_ layers. (b) DFT calculated relative total energies of fully strained RuO_2_/TiO_2_ superlattices (*U* = 0) for uncompensated antiferromagnetic (AFM; weak ferromagnetic), ferromagnetic (FM), and nonmagnetic (NM) states. The AFM (weak FM) configuration is energetically favored. (c) Magnetic anisotropy energy of the AFM (weak FM) state as a function of the spin‐axis orientation between the [001] and [110] crystallographic directions, identifying [001] as the magnetic easy axis. (d) Atom‐resolved spin‐density distribution for the RuO_2_/TiO_2_/RuO_2_ heterostructure (schematically shown on the left), highlighting Ru‐derived magnetic moments. (e) Projected magnetic moment profile plotted across the atomic rows of the heterostructure in (d).

DFT calculations on RuO_2_/TiO_2_ superlattices reveal net magnetization values that agree closely with the PNR results (Figure 3). Figure 3a,b summarize the key quantities extracted from experiment and theory, respectively. To quantitatively assess uncertainties and establish confidence intervals, we performed the DREAM Markov‐chain Monte Carlo (MCMC) algorithm implemented in the bumps Python package (Table S1) [50, 51, 57]. Importantly, the sampling window explicitly included zero‐net magnetization solutions, enabling rigorous statistical comparison between magnetic and non‐magnetic models within the Bayesian framework. The net magnetization values obtained from PNR experiments are (0.0219 ± 0.0039) and (0.0111 ± 0.0080) *µ*_B_ f.u.^−1^ for the [2.8 nm RuO_2_|5.6 nm TiO_2_]_5_​ and [1.8 nm RuO_2_|6.9 nm TiO_2_]_5_ ​superlattices, respectively (Figure 3a). These results support the presence of an uncompensated magnetization along the [001] direction of the fully strained RuO_2_ layers of the superlattices, beyond the bounds of the fitting uncertainty. It is worth comparing our results with the expected magnetic field‐induced paramagnetic moments. Assuming a molar susceptibility of RuO_2_ of 1.5 × 10^−4^ emu mol^−1^ Oe^−1^ at 4 K [3], the moment induced by a 4.8 T magnetic field is only ∼0.0013 *µ*_B_ f.u.^−1^. Thus, the net moment we observe along the [001] direction cannot be explained by paramagnetism alone. For the DFT calculations, we adopted a fully strained [2 nm RuO_2_|2 nm TiO_2_]_5_ superlattice without a Hubbard *U* correction (*U* = 0). The calculated total magnetization of the uncompensated AFM (weak FM) state is 0.0288 *µ*_B_ f.u.^−1^, in good quantitative agreement with the PNR values, whereas the FM state yields a much larger moment of 0.2820 *µ*_B_ f.u.^−1^ (left panel of Figure 3b).

The relative total energies of the AFM (weak FM), FM, and nonmagnetic (NM) configurations (right panel of Figure 3b) establish the AFM state as the magnetic ground state (the lowest energy), consistent with earlier RuO_2_ studies [12, 21, 22, 23, 24]. We further note that the experimentally extracted magnetization values also rule out the FM state (Figure S6b). These results align with recent observations in fully strained RuO_2_/TiO_2_ (110) films, including spin‐split bands in spin‐ARPES [12], time‐reversal symmetry breaking in optical second‐harmonic generation [21], and anomalous Hall effect [23], and recent first‐principles predictions of strain‐stabilized magnetic order [22, 58]. In contrast, several recent studies reporting markedly different conclusions were conducted on strain‐relaxed thicker RuO_2_/TiO_2_ films (typically ≥ 5–30 nm) [9, 53, 59] where partial strain relaxation, variations in oxygen stoichiometry, and differences in measurement sensitivity may substantially alter the observed magnetic response. Group theory analysis further indicates that the uncompensated magnetic phase hosts a coexistence of weak FM and altermagnetism [21, 60], while the theoretical calculations reveal a non‐collinear spin texture, in which neighboring spins are not strictly parallel or antiparallel, carrying both octupolar (AFM/altermagnetic) and non‐zero net magnetic (weak FM) moments [21]. While conventional PNR is insensitive to fully compensated magnetic states—where opposing magnetic moments cancel each other out perfectly (e.g., in a bulk antiferromagnet) [61]—it possesses high sensitivity to weak uncompensated moments—making it ideally suited for detecting and quantifying magnetic states stabilized in our strained RuO_2_ layers within the superlattice.

Magnetic anisotropy energy calculations indicate that the magnetic easy axis lies along the in‐plane [001] direction, rather than the out‐of‐plane [110] direction (Figure 3c). The atom‐resolved spin‐density distribution (Figure 3d) shows that the magnetic moments originate predominantly from Ru atoms, consistent with the layer‐resolved magnetic SLD obtained from PNR analysis (Figure 2d). Notably, the projected magnetic moment profile in Figure 3e reveals a pronounced interfacial enhancement of the magnetic order near the RuO_2_/TiO_2_ boundaries, which might be related to the interfacial magnetic features observed in the [1.8 nm RuO_2_|6.9 nm TiO_2_]_5_ superlattices (Figure S8). This feature is reminiscent of the theoretically predicted interfacial magnetic behavior at the boundary between the dielectric SrTiO_3_ and the spin‐polarized metal SrRuO_3_ [62].

## 3 Conclusion

In summary, our results directly observed the magnetic order in RuO_2_/TiO_2_ superlattices via PNR under field‐cooled conditions, combined with first‐principles calculations. We identify (1) a strain‐stabilized magnetic state associated with the epitaxial strain, and (2) the uncompensated magnetic order arising from strain‐induced crystal distortions. Our approach targets the uncompensated magnetic component using PNR, providing direct evidence for a net magnetization under field‐cooled conditions and addressing the key aspect of the ongoing debate regarding the magnetic state of strained RuO_2_. Importantly, even the small uncompensated moment detected here serves as a direct signature of the underlying symmetry‐broken magnetic state and its associated spin‐dependent electronic functionalities. These results establish epitaxial strain as a powerful and versatile control knob for engineering, accessing, and controlling magnetic phases in correlated oxides.

## 4 Methods

### 4.1 Polarized Neutron Reflectometry

PNR experiments were performed on the Magnetism Reflectometer MAGREF at the Spallation Neutron Source at Oak Ridge National Laboratory [63, 64, 65, 66], using neutrons with wavelengths λ in the range of 0.2–0.8 nm and a high degree of polarization of 98.5–99%. The measurements were performed in a closed‐cycle refrigerator equipped with a 5 T cryomagnet (Cryomagnetics). Using the time‐of‐flight method, a beam of collimated polychromatic polarized neutrons with a wavelength band of δλ impinged onto a film at a grazing angle θ, interacting with atomic nuclei and the spins of unpaired electrons. The reflected intensities R+ and R− were measured as a function of momentum transfer, *q*_110_ = 4π sin(θ)/λ, with the neutron spin parallel (+) or antiparallel (−) to the applied field, respectively. To separate nuclear and magnetic scattering, the spin asymmetry ratio SA = (R^+^(*q*_110_) − R^−^(*q*_110_))/(R^+^(*q*_110_) + R^−^(*q*_110_)) was calculated, where SA = 0 corresponds to a zero magnetic moment in the system. Being electrically neutral, spin‐polarized neutrons penetrate the entire multilayer structures and probe the magnetic and structural composition of the film and buried interfaces all the way down to the substrate.

### 4.2 Superlattice Growth

RuO_2_/TiO_2_ (110) superlattices were synthesized on TiO_2_ (110) single crystal substrates (Crystec) using a hybrid molecular beam epitaxy (MBE; Scienta Omicron). Substrates were cleaned sequentially with acetone, methanol, and isopropanol, followed by a 2‐h bake at 200°C in the load‐lock chamber. Before growth, substrates were annealed for 20 min in an oxygen plasma at 300°C to ensure a clean surface. The thin TiO_2_ buffer layer was incorporated to avoid substrate‐induced variations and ensure consistent growth across different samples [23]. Each RuO_2_/TiO_2_ superlattice period was fabricated by alternating the cell shutter control of RuO_2_ and TiO_2_ layers. RuO_2_ layers were grown using thermally evaporated Ru(acac)_3_ delivered from a low‐temperature effusion cell (MBE Komponenten) operated at 170–180°C. TiO_2_ layers were grown by using titanium tetraisopropoxide (TTIP, 99.999%, Sigma–Aldrich) injected through a line‐of‐sight gas injector (E‐Science Inc.) controlled via a customized linear‐leak valve system with a Baratron capacitance manometer (MKS Instruments Inc.). All growth steps were performed under radio‐frequency oxygen plasma at a pressure of 5 × 10^−6^ Torr. To suppress oxygen vacancy formation, the samples were cooled to 120°C in oxygen plasma after completing the superlattice growth. Real‐time growth evolution of surfaces was monitored through reflection high‐energy electron diffraction (RHEED) before, during, and after growth.

### 4.3 Structural Characterization

Structural properties of the RuO_2_/TiO_2_ superlattices–including crystallinity, superlattice periodicity, thickness, interface sharpness, and strain state–were characterized using high‐resolution x‐ray diffraction (XRD; Rigaku SmartLab XE) *θ–*2*θ* scans and x‐ray reflectivity (XRR; Rigaku SmartLab XE) were used to determine the superlattice period and interfacial roughness, while reciprocal space maps (RSMs) around asymmetric reflections were used to quantify the in‐plane and out‐of‐plane strain states. All XRD measurements were performed using Cu Kα radiation operated at 40 kV and 35 mA. Surface morphology was examined via atomic force microscopy (Bruker Nanoscope V Multimode 8) operated in peak‐force tapping mode.

### 4.4 Density Functional Theory

We performed first‐principles calculations based on density functional theory (DFT) as implemented in the Vienna *ab‐initio* simulation package (VASP) [67, 68]. We employed the projector augmented wave (PAW) method [69, 70] and the Perdew–Burke–Ernzerhof (PBE) potential [71]. The kinetic energy cutoff of electronic wavefunctions was set to 500 eV. To describe (110)‐oriented RuO_2_, we aligned three lattice vectors of RuO_2_ along [001], [$\bar{1}$10], and [110] directions, respectively. The theoretical equilibrium lattice constants of bulk RuO_2_ and TiO_2_ were calculated to be $a_{\left[001\right]}^{RuO_{2}}=3.119Å,anda_{\left[\bar{1}10\right]}^{RuO_{2}}=a_{\left[110\right]}=6.394Å$,  and $a_{\left[001\right]}^{TiO_{2}}=2.970Å,anda_{\left[\bar{1}10\right]}^{TiO_{2}}=a_{\left[110\right]}=6.588Å$, respectively. For the RuO_2_/TiO_2_ superlattice structure, we constructed a supercell structure including six atomic sublayers of RuO_2_ and six atomic sublayers of TiO_2_, respectively. The TiO_2_ thickness in the DFT model was reduced to keep the computational cost tractable while retaining the essential strained heterostructure. The in‐plane lattice constants of the supercell are fixed to those of bulk TiO_2_, while the out‐of‐plane lattice constants are fully relaxed, yielding a value of 39.5 Å. This fully strained configuration is consistent with the absence of strain relaxation observed in the experimental XRD RSM measurements for reduced TiO2 thicknesses [21]. The corresponding Brillouin zone of the supercell was sampled using an 8 × 4 × 1 k‐grid mesh. Spin–orbit coupling was included only in the calculation of the magnetic anisotropy energy. Previous calculations on fully strained RuO_2_ further showed that the noncompensated antiferromagnetic state remains energetically favorable over the ferromagnetic and nonmagnetic states for finite *U* [22, 23]. In the present study, we therefore used *U* = 0 to minimize the number of adjustable parameters in the DFT calculations.

### 4.5 X‐ray Photoelectron Spectroscopy

Film composition was analyzed using x‐ray photoelectron spectroscopy (XPS; PHI 5000 VersaProbe III Photoelectron Spectrometer) with an Al K*α* source (1486.6 eV) and settings including an energy step size of 0.05 eV and a pass energy of 55 eV. A flood gun was adopted to prevent photoemission‐induced surface charge effects. All spectra were calibrated using the binding energy of C─C bonds (284.8 eV) from the adventitious carbon on the surface.

### 4.6 Magnetization Measurement

Magnetic properties were characterized using a SQUID (Superconducting Quantum Interference Device) magnetometer (Quantum Design MPMS3) with the magnetic field applied along the in‐plane [001] direction. M‐H loops were collected between −5 and +5 T at 2, 50, and 400 K for both the [2 nm RuO_2_|1 nm TiO_2_]_5_ superlattice and a TiO_2_ reference film grown under the same growth conditions using hybrid MBE.

## Conflicts of Interest

The authors declare no conflicts of interest.

## Supporting information




**Supporting File**: advs77914‐sup‐0001‐SuppMat.pdf.

## Data Availability

The data that support the findings of this study are available in the Supplementary Material of this article.
