# Supplementary material for: Emergence of Unconventional Magnetic Order in Strain‐Engineered RuO2/TiO2 Superlattices
Source: Adv Sci (Weinh). 2026 Sep 27:e77914. Online ahead of print. doi: 10.1002/advs.77914 (PMC13616356; doi:10.1002/advs.77914)
Supplement: Supplementary file 1 [file ADVS-9999-e77914-s001.pdf]

Supporting Information

Emergence of unconventional magnetic order in strain-engineered RuO_2/TiO_2 superlattices

Seung Gyo Jeong, Seungjun Lee, Jin Young Oh, Bonnie Y.X. Lin, Anand Santhosh, Sehwan Song, Sungkyun Park, James M. LeBeau, Alexander J. Grutter, Woo Seok Choi, Tony Low, Valeria Lauter*, and Bharat Jalan**

Table of Contents

Figures S1 to S9

Table S1

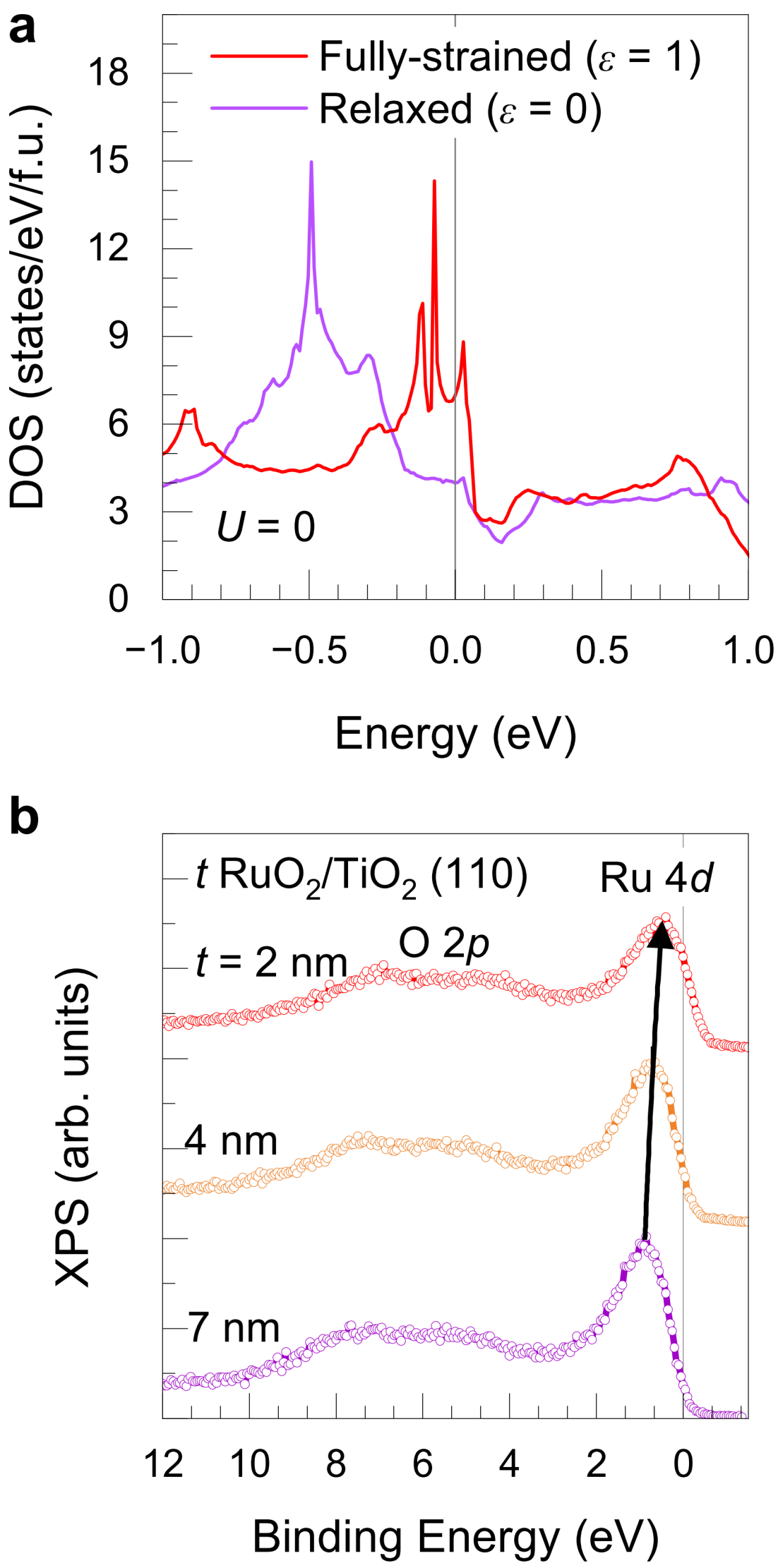

Figure S1. Strain-induced modulation of electronic structure. (a) Projected Ru 4*d* density of states (DOS) calculated for the fully strained ($\varepsilon = 1$) and relaxed ($\varepsilon = 0$) RuO_2 structures, showing a substantial enhancement of spectral weight near the Fermi level under strain. (b) X-ray photoelectron spectra (XPS) valence band spectra of RuO_2/TiO_2 (110) thin films with varying RuO_2 thickness (*t*). The systematic enhancement of spectral intensity near the Fermi level is consistent with the strain-modulated DOS predicted by theoretical calculations. The arrow represents a guide to the eye.

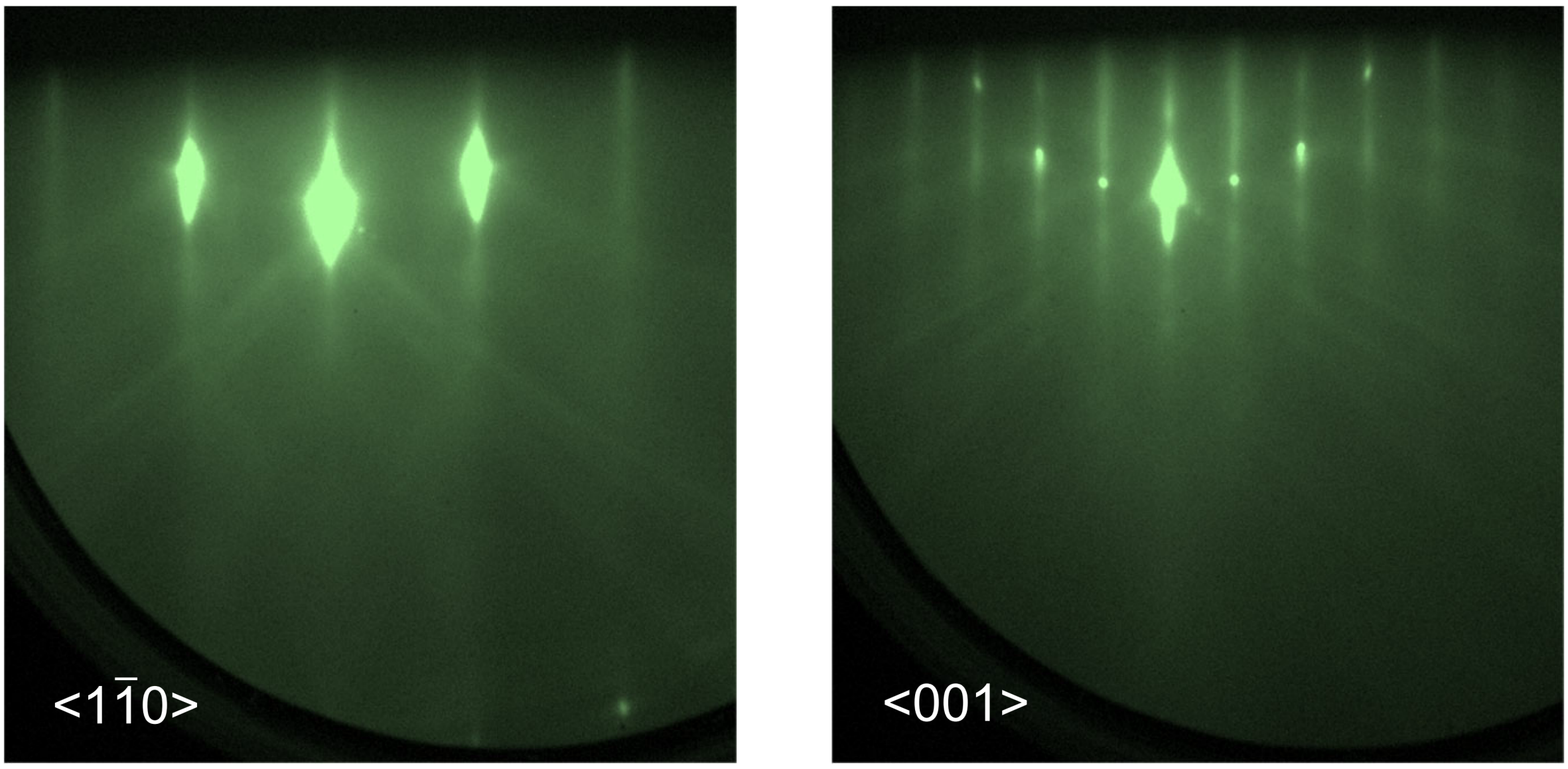

Figure S2. Reflection high-energy electron diffraction (RHEED) patterns after growth for [1.8 nm RuO_2|6.9 nm TiO_2]$_5$ show the streaky RHEED patterns along both in-plane crystallographic orientations.

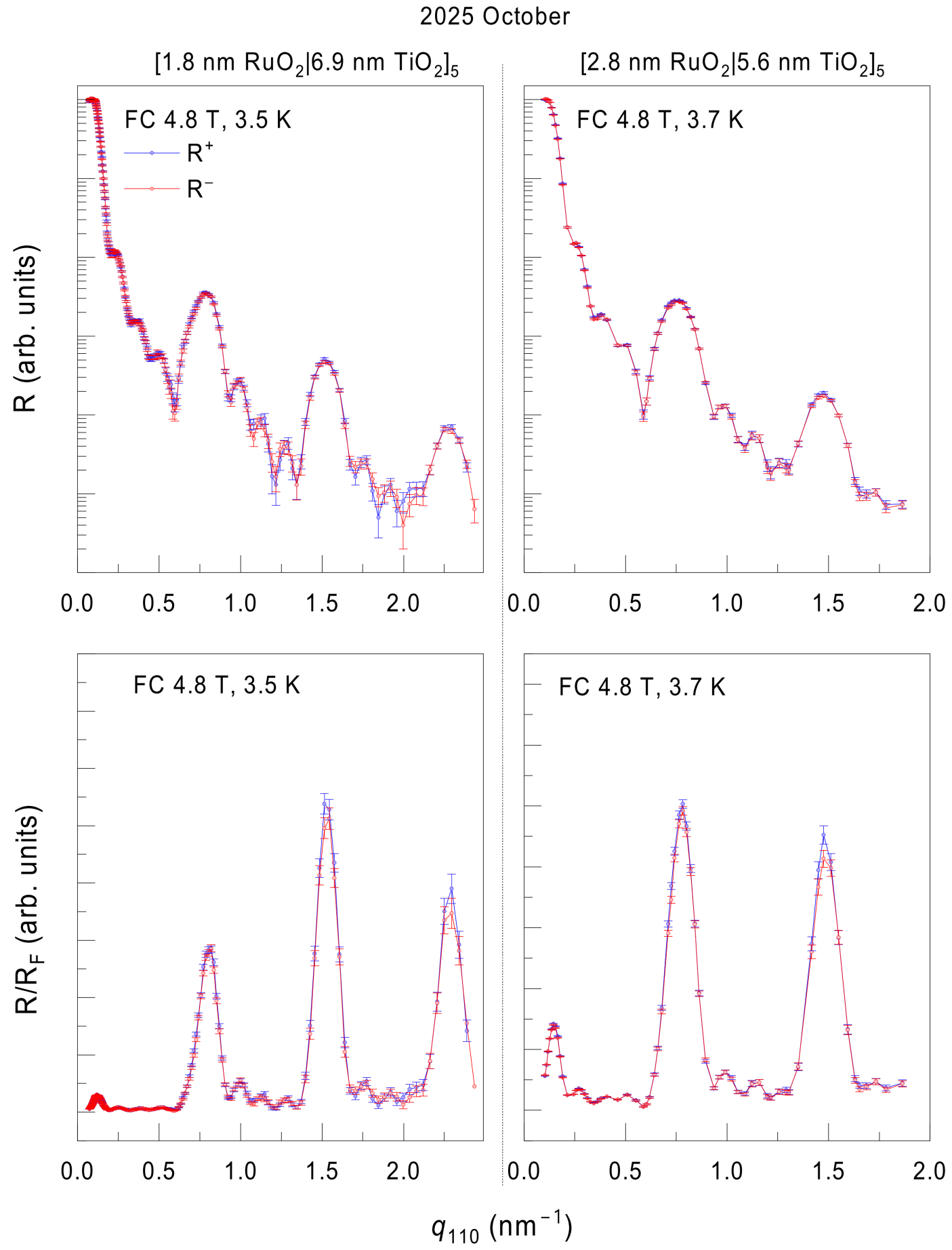

Figure S3. PNR results for two different RuO_2/TiO_2 superlattice samples ([1.8 nm RuO_2|6.9 nm $TiO_2]_5$ and [2.8 nm RuO_2|5.6 nm $TiO_2]_5$) measured with spin-up (R^+) and spin-down (R^-) polarization channels. The bottom panels show the Fresnel-normalized reflectivity, plotted as $R(q_{110})q_{110}^4$. Both datasets were measured within a single beamtime session in October 2025.

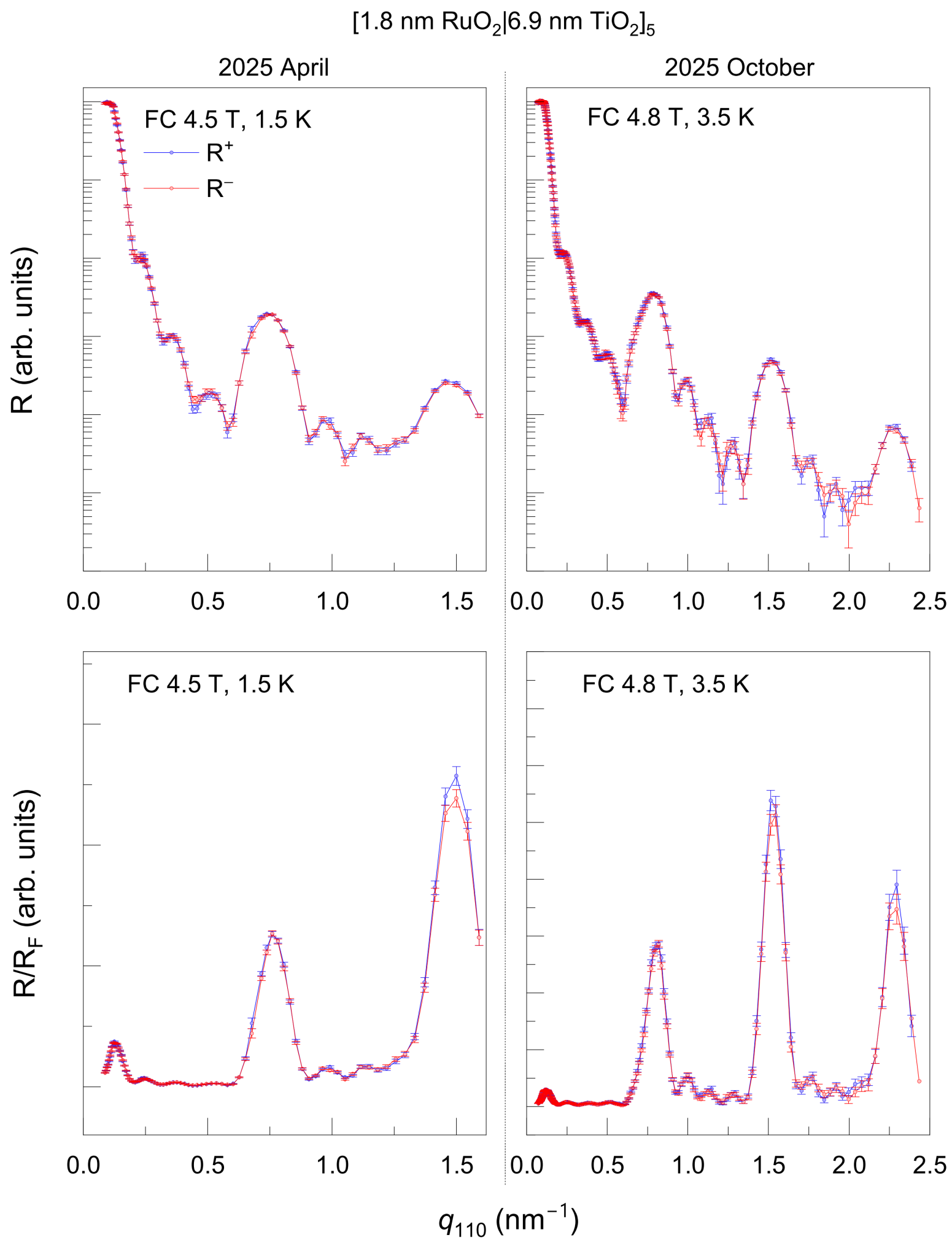

Figure S4. PNR results for the same [1.8 nm RuO_2|6.9 nm TiO_2]$_5$ superlattice sample obtained in two separate beamtime sessions. Both measurements consistently show a small but robust magnetic signal near the superlattice peaks.

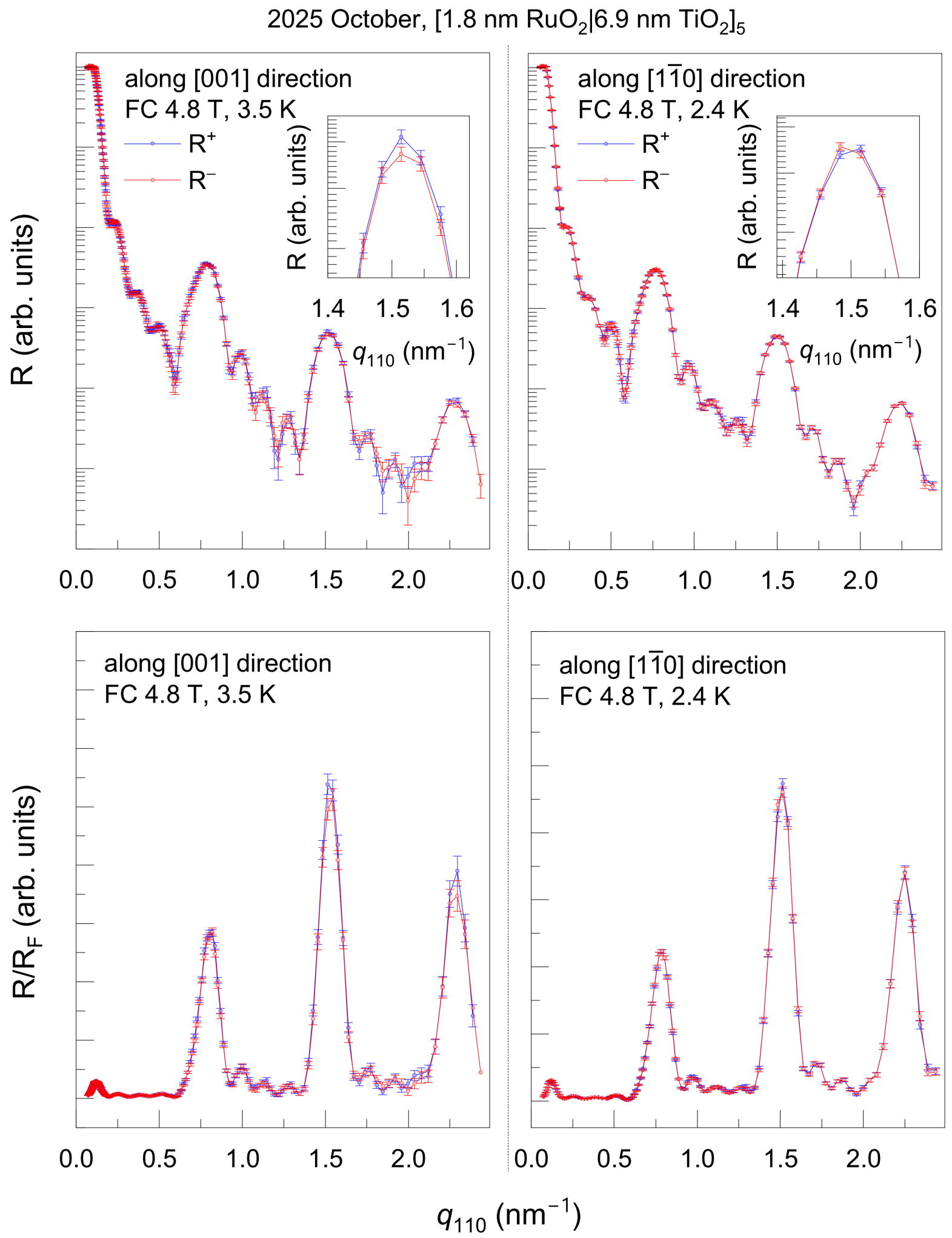

Figure S5. PNR results for the [1.8 nm RuO_2|6.9 nm TiO_2]$_5$ superlattice obtained under two different in-plane geometries. In these measurements, both the applied magnetic field and the incident neutron beam were aligned along two different in-plane crystallographic orientations, [001] (left) and [$1\bar{1}0$] (right). A magnetic signal near the superlattice peaks is observed only when the measurement is performed along the [001] orientation.

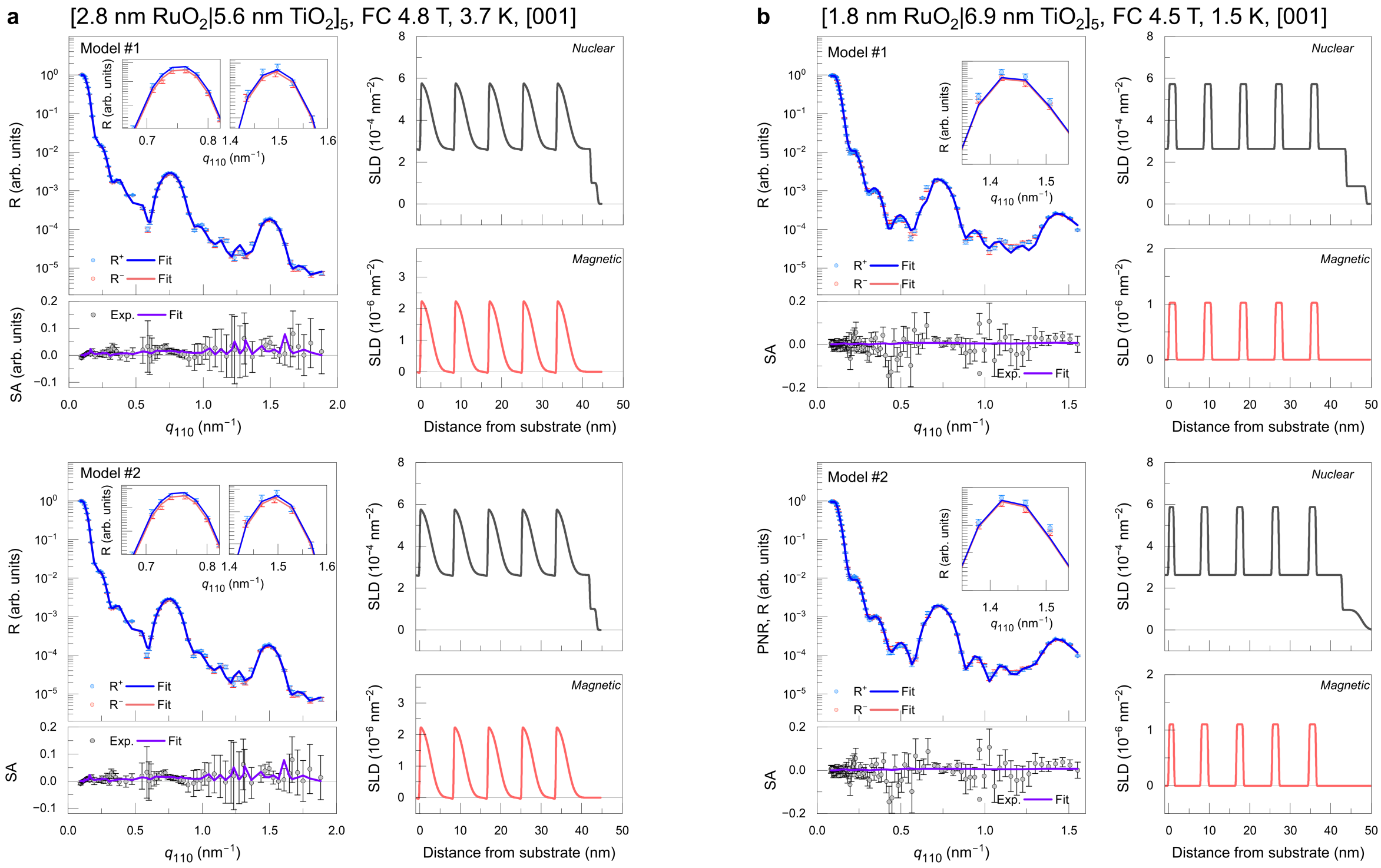

Figure S6. The PNR fitting results. (a) PNR results for [2.8 nm RuO_2|5.6 nm TiO_2]$_5$ (April 2025) and (b) [1.8 nm RuO_2|6.9 nm TiO_2]$_5$ (October 2025) superlattices measured with spin-up (R^+) and spin-down (R^-) polarization channels, with the fitting results and depth profiles of the nuclear and magnetic SLD. The corresponding model fits, and the extracted depth profiles of the nuclear and magnetic SLDs are shown. Details of the fitting models, parameter results, and χ^2 values are provided in Table S1.

Samples	Measurement directions	Markov-Chain Monte Carlo (MCMC) simulations		
		Model	Magnetization (μ_B f.u.$^{-1}$)	χ^2
[2.8 nm RuO_2|5.6 nm TiO_2]$_5$	[001]	#1	0.0233 ± 0.0040	9.87
		#2	0.0219 ± 0.0039	2.50
	[1$\bar{1}$0]	#1	0.0058 ± 0.0030	19.26
		#2	0.0031 ± 0.0019	8.57
[1.8 nm RuO_2|6.9 nm TiO_2]$_5$	[001]	#1	0.0103 ± 0.0060	7.85
		#2	0.0111 ± 0.0080	2.37

Table S1. Summary of Markov-Chain Monte Carlo (MCMC) analysis results. Uncertainty analyses were carried out using the Refl1D software in combination with the DREAM MCMC algorithm implemented in the bumps Python package. Two structural models were evaluated: (1) a uniform-thickness model in which all RuO_2 layers were constrained to have identical thickness, and (2) a variable-thickness model allowing individual RuO_2 layer thicknesses to vary within ~1 nm independently. All TiO_2 layers were constrained to be identical, except for the topmost layer, for which the surface roughness was allowed to differ. Corresponding fitting results for PNR data performed along the [001] direction are presented in Figure S6. The χ^2 values obtained for model #2 are systematically lower, indicating the presence of slight RuO_2 layer-to-layer variations. Importantly, both modeling approaches consistently yield a robust uncompensated magnetization along the [001] direction within tight confidence intervals.

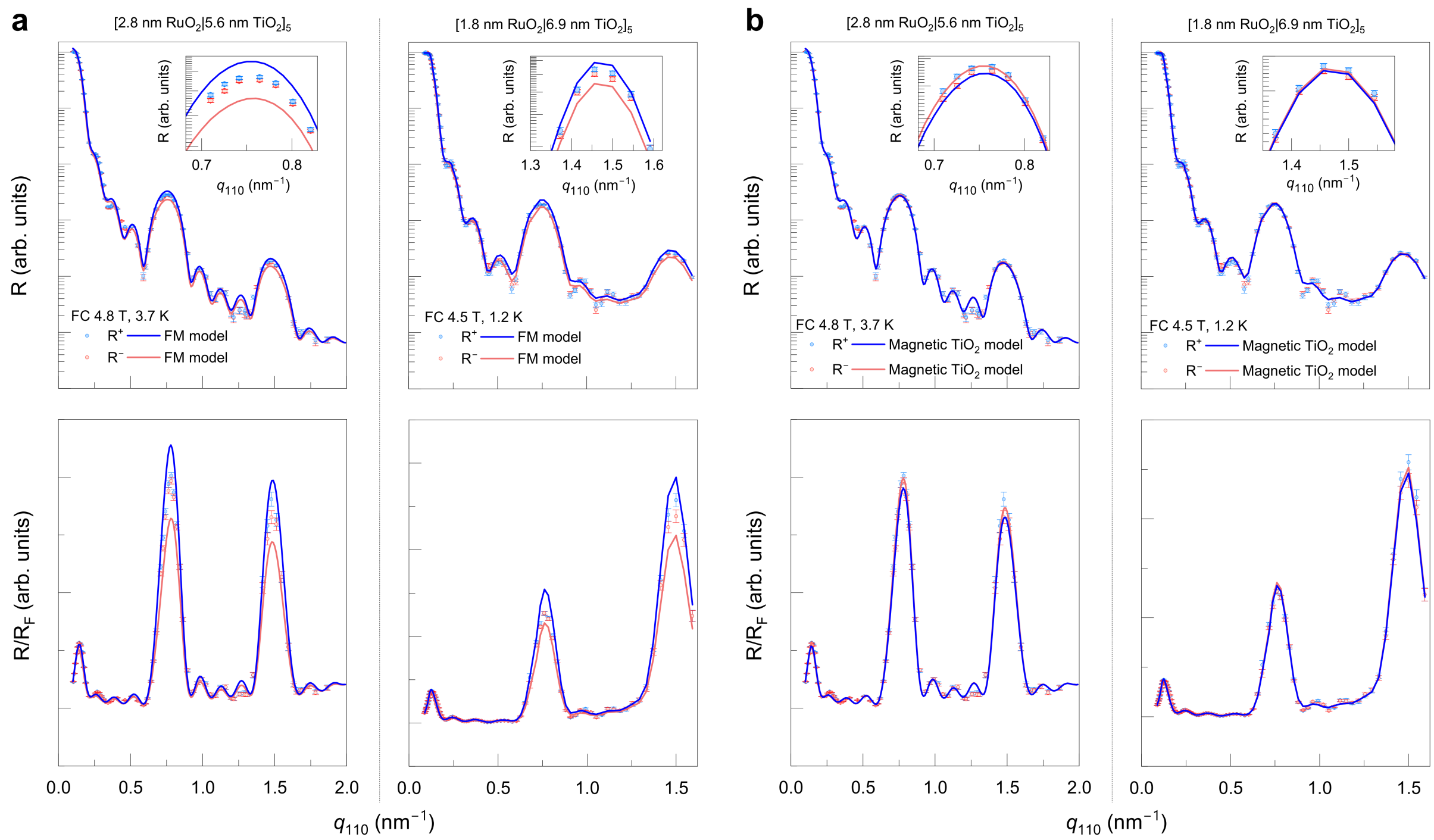

Figure S7. PNR simulation and data comparisons for [2.8 nm RuO_2|5.6 nm TiO_2]$_5$ and [1.8 nm RuO_2|6.9 nm TiO_2]$_5$ superlattices with a, conventional ferromagnetic (FM) RuO_2 model and b, magnetic TiO_2 model. For the conventional FM model, we applied the FM magnetic moments as a 0.2820 μ_B f.u.$^{-1}$ predicted from the DFT calculations. For the magnetic TiO_2 model, we applied the magnetic moments in TiO_2 layers as 0.037 and 0.024 μ_B f.u.$^{-1}$ for [2.8 nm RuO_2|5.6 nm TiO_2]$_5$ and [1.8 nm RuO_2|6.9 nm TiO_2]$_5$ superlattices, respectively.

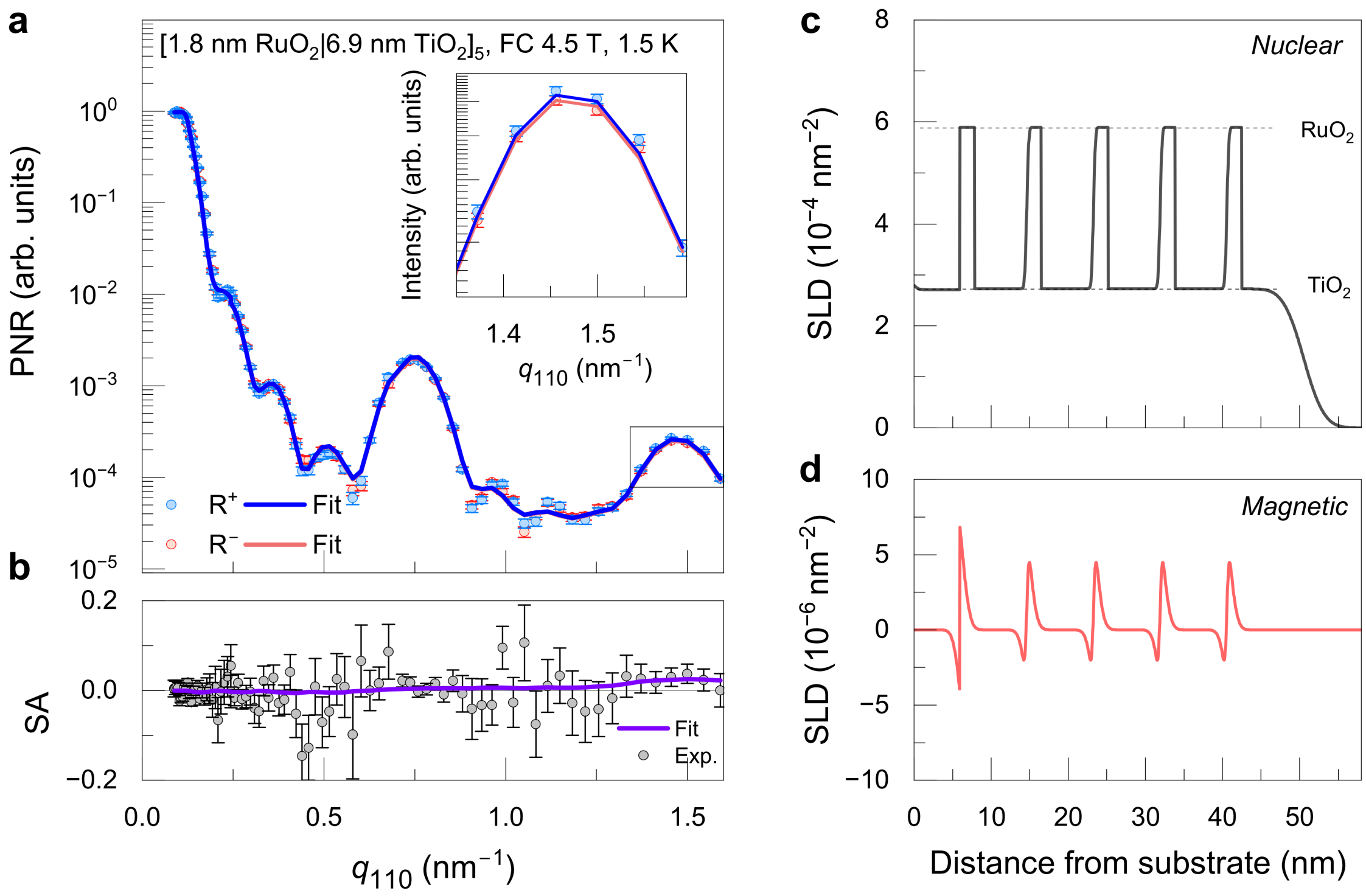

Figure S8. PNR results for [1.8 nm RuO_2|6.9 nm TiO_2]$_5$ superlattice. (a) PNR results for [1.8 nm RuO_2|6.9 nm TiO_2]$_5$ superlattice measured with spin-up (R^+) and spin-down ($R^–$) polarization channels, together with the fitting results. This measurement was performed with a 4.5 T magnetic field at 1.5 K in April 2025. (b) Spin asymmetry (SA) is defined as ($R^+(q_{110})$ − $R^-(q_{110})$)/($R^+(q_{110})$ + $R^-(q_{110})$), along with the fits. (c) Depth profiles of the nuclear and (d) magnetic SLDs used in reflectivity modeling. The horizontal dashed lines in the SLD represent guides to the eye. Notably, the larger spin splitting observed at the second superlattice Bragg peak compared to the first suggests the presence of interfacial magnetizations. To capture this effect, each RuO_2 layer was divided into four sublayers, with their magnetization and roughness values fitted independently. In the fitting procedure, we employed log(FOM) to ensure balanced sensitivity across a wide dynamic range, which is particularly important for superlattice peaks where the reflectivity spans several orders of magnitude. The average magnetization within the RuO_2 layers is estimated to be 0.024 μ_B f.u.$^{-1}$.

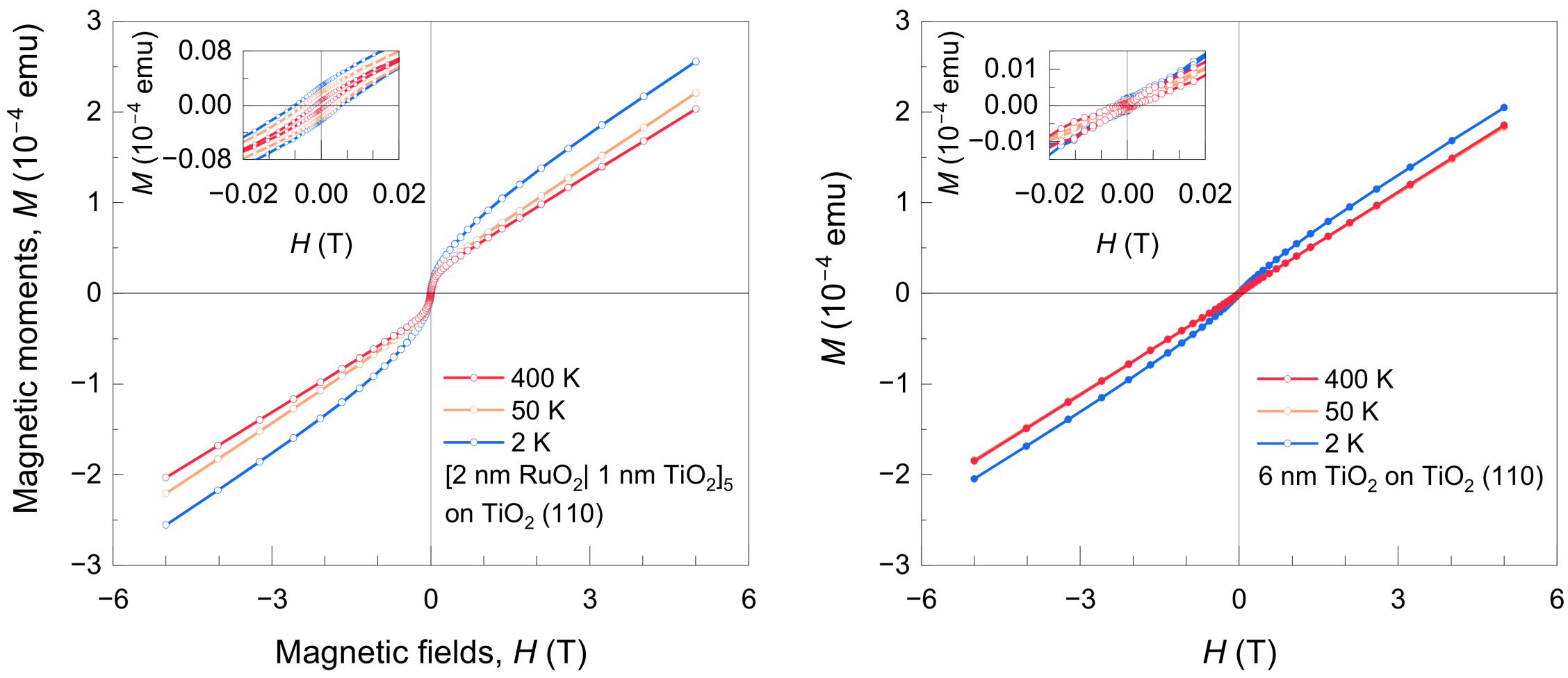

Figure S9. SQUID magnetometry of RuO_2/TiO_2 superlattices. Magnetic hysteresis loops measured with the magnetic field applied along the [001] direction for (left) a [2 nm RuO_2 | 1 nm TiO_2]$_5$ superlattice and (right) a 6 nm thick TiO_2 reference film on TiO_2 (110). Data were collected at 2, 50, and 400 K. Insets show enlarged views near zero field. A finite hysteresis is observed only in the superlattice, indicating weak ferromagnetic ordering consistent with the uncompensated magnetic moment determined from PNR. The SQUID data shown were not corrected for the TiO_2 substrate or background contribution.
